# Blocking Honeybee Preference for Neonicotinoids by Interfering With Dopamine Signaling

**DOI:** 10.1002/advs.78535

**Published:** 2026-11-04

**Authors:** Jing Li, Yuan Ge, Wangjiang Feng, David C. Lyon, Pingli Dai, Yong‐Jun Liu

**Affiliations:** ^1^ State Key Laboratory of Resource Insects Institute of Apicultural Research Chinese Academy of Agricultural Sciences Beijing China; ^2^ State Key Laboratory of Regional and Urban Ecology Research Center for Eco‐Environmental Sciences Chinese Academy of Sciences Beijing China; ^3^ University of Chinese Academy of Sciences Beijing China; ^4^ Department of Anatomy and Neurobiology School of Medicine University of California Irvine California USA

**Keywords:** dopamine, honeybee, neonicotinoids, preference, thiamethoxam

## Abstract

Honeybees (*Apis mellifera*) prefer nectar containing neonicotinoids despite severe exposure risk leading to colony collapse. The neural mechanisms underlying this preference behavior remain unknown, impeding the development of effective blocking strategies. Neonicotinoids act as acetylcholine receptor agonists, and dopamine is a vital neuromodulator in addictive behavior. We hypothesize that neonicotinoids could activate brain dopamine signaling in honeybees, reinforcing their feeding behavior. Here, we show that thiamethoxam‐laden food is preferred over uncontaminated options, and that prolonged consumption is associated with transient, state‐dependent increases in brain dopamine concentrations. Introducing the dopamine receptor antagonist, fluphenazine, reduces thiamethoxam preference. Our study first reveals a key dopamine‐associated mechanism underlying neonicotinoid‐induced preference behavior in honeybees and provides a potential basis for future strategies to reduce honeybee attraction to insecticide‐containing food source through modulation of the preference‐related dopamine pathway, thereby helping to preserve honeybee pollination services.

## Graphical abstract

Honeybees (*Apis mellifera*) paradoxically prefer nectar containing the harmful neonicotinoid thiamethoxam. This preference is associated with transient brain dopamine elevation during proboscis extension reflex (PER) responses and active feeding, and is reduced by the dopamine receptor antagonist fluphenazine. These findings provide a basis for future strategies to reduce honeybee attraction to neonicotinoid‐laden nectar, thereby helping preserve pollination services.

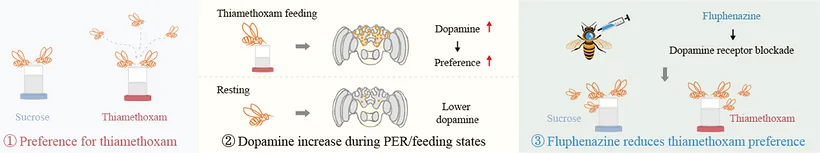

## 1 Introduction

Honeybees (*Apis mellifera*) are important pollinators, playing crucial roles in agricultural productivity and biodiversity conservation [1, 2]. Over 90% of the 107 major crops worldwide rely on their pollination, and without these services, crop yields could decrease by 5%–8% [3]. Neonicotinoids, which account for approximately 30% of the global insecticide market [4], are extensively used in agriculture and pose long‐term risks to the environment and to honeybees. A global analysis of honey samples found that 75% contained at least one neonicotinoid compound [5], indicating widespread insecticide exposure of honeybees. Although honeybee colony declines result from multiple interacting factors, including parasites, pathogens, habitat loss, and nutritional stress [6], neonicotinoid exposure has been recognized as an important stressor affecting honeybee health [7, 8]. Field monitoring studies have detected thiamethoxam residues of 3.26 ng g^−1^ (11.18 nM) in pollen and 3.20 ng g^−1^ (10.97 nm) in nectar [9], which are comparable to the environmentally relevant concentration range (1–10 nm) reported by Kessler et al. [10].

Previous studies have shown that exposure to sublethal concentrations of neonicotinoids impairs learning, memory, and motor function in honeybees [11], reduces pollination and foraging efficiency [11], and decreases survival and reproductive rates [12, 13, 14], eventually leading to colony collapse. Given these adverse effects, one might expect forager honeybees to avoid nectar or pollen containing neonicotinoids to minimize exposure risks. However, contrary to this expectation, recent studies reveal that honeybees do not avoid neonicotinoid‐laden nectar; instead, they exhibit a feeding preference for it, particularly for the neonicotinoid insecticide thiamethoxam [10]. Given the high foraging demands to sustain the colony, this preference leads to increased intake of neonicotinoid‐laden food to the hive, thereby posing a serious threat to in‐hive honeybees, and to human health due to the accumulation of insecticide residues in apicultural products, e.g., honey, beeswax, and propolis. While the behavioral preference for neonicotinoid‐laden nectar is well‐documented, whether honeybees can detect neonicotinoid‐containing food through sensory pathways during food selection and what mechanisms drive neonicotinoid‐preferred behavior in honeybees remain unclear. Understanding these mechanisms is critical for exploring how neonicotinoids affect the honeybee nervous system and may contribute to the development of innovative withdrawal treatments to block this neonicotinoid preference behavior, ultimately reducing exogenous insecticide risks to honeybee colonies and human health.

Neonicotinoids are insecticides targeting insect nicotinic acetylcholine receptors (nAChRs, nerve signal‐receiving proteins), mimicking the effect of the neurotransmitter acetylcholine, and thus disrupting central nervous system signaling to induce insect convulsions, paralysis, and death [15, 16]. As a result, similar to the addictive effects of nicotine, neonicotinoids may stimulate dopamine release in the brain dopamine system [17, 18], which is implicated in food wanting [19], potentially interfering with the reward pathway and reinforcing feeding preference [20]. Therefore, we hypothesized that this positive feedback is mediated by rapidly enhance brain dopamine signaling after oral ingestion of neonicotinoids, inducing increased feeding preference for neonicotinoid‐laden food in honeybees.

To test this hypothesis, we monitored the feeding preference of honeybees for thiamethoxam, detected the sensory recognition pathways, and quantified and blocked brain dopamine. We found that honeybees tend to feed on foods containing thiamethoxam, independent of olfactory recognition, accompanied by significantly elevated brain dopamine levels. Moreover, by blocking dopamine signaling, thiamethoxam preference and consumption were significantly reduced. Our study first demonstrates a dopamine‐driven mechanism underlying neonicotinoid preference behavior in honeybees, and provides a feasible therapy to mitigate the risk of neonicotinoid exposure, thereby preserving honeybee and human health.

## 2 Results and Discussion

### 2.1 Honeybees Prefer to Consume Foods Containing Thiamethoxam

Many studies have shown that acute or chronic exposure to thiamethoxam can negatively affect honeybee survival [21, 22]. Accordingly, it is essential first to establish a range of thiamethoxam exposure levels expected to have no significant impact on honeybee survival. Honeybees were orally exposed to thiamethoxam at concentrations of 0 (Control, CK), 1, 5, 10, 15, 30, and 40 nm, corresponding to approximately 0, 0.29, 1.46, 2.92, 4.38, 8.76, and 11.68 ng g^−1^, respectively, along with a dimethyl sulfoxide (DMSO) solvent control (SC), for 10 days (see Section 4.1 and 4.3). No significant differences were observed between the CK and SC groups in either survival (Figure S1A, *P*_SC_ > 0.05) or body weight (Figure S1B, *P*_SC_ > 0.05), indicating that the solvent control DMSO did not affect honeybee survival or body weight. Therefore, the CK group was used as the reference control for subsequent comparisons. Compared with the CK group, honeybee survival was significantly reduced after exposure to thiamethoxam concentrations above 10 nm for 10 days (Figure S1A, *P*_15 nM_ < 0.01, *P*_30 nM_ < 0.001, *P*_40 nM_ < 0.001), and 40 nm thiamethoxam caused a significant reduction in honeybee body weight (Figure S1B, *P*_40 nM_ < 0.05). These results suggest that long‐term exposure to thiamethoxam concentrations above 10 nm significantly impairs honeybee health. Therefore, we selected 1, 5, and 10 nm of thiamethoxam to further explore the feeding preference of honeybees, and these concentrations were consistent with thiamethoxam environmental residues commonly found in nectar and pollen [9].

The feeding preference of honeybees for thiamethoxam‐laden food at these lower concentrations was demonstrated by two methods. First, we tested feeding preference under restricted laboratory conditions where newly emerged honeybees were group‐ housed in separate cages. Each cage was exclusively provided with one of five sucrose solutions: either one of the two controls (CK, SC) or one of the three concentrations of thiamethoxam (1, 5, and 10 nm; Figure 1A, see Section 4.4). During the experimental period, no pollen was provided to any treatment group. We found that the cumulative intake of sucrose solutions containing thiamethoxam (> 1 nm) over the 10 days was significantly higher than in the CK group (Figure 1B, *P*_5 nM_ < 0.001, *P_10_*_nM_ < 0.001), indicating honeybees are willing to consume more thiamethoxam‐laden food even at low concentrations.

**FIGURE 1:**
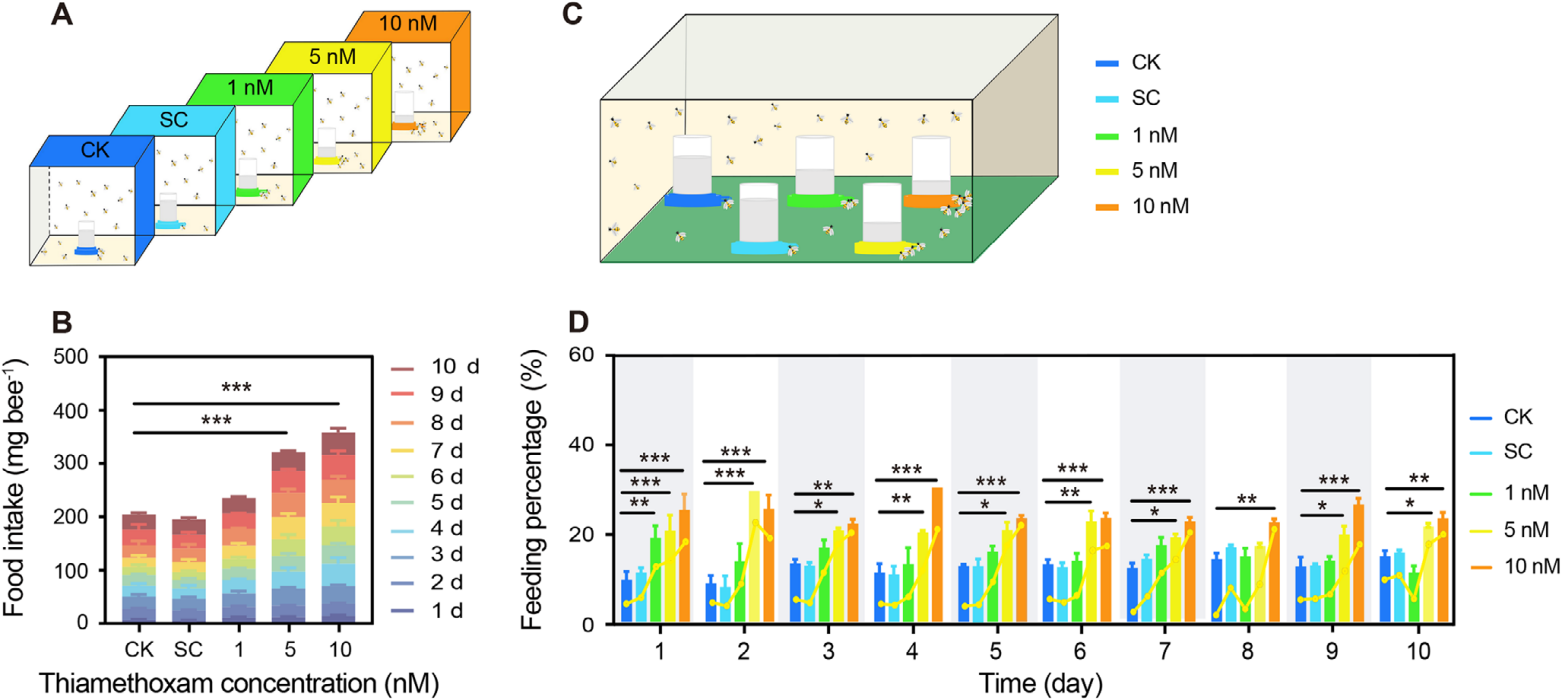
Honeybees show a preference for consuming thiamethoxam‐laden food. (A) Schematic diagram of the fixed‐choice assay. Honeybees were group‐housed, with each cage provided exclusively with one of the five sucrose solutions: Control (CK), solvent control (SC), and thiamethoxam solutions at concentrations of 1, 5, and 10 nm. (B) During the 10 days in the fixed‐choice assay, the cumulative food intake was significantly higher in the 5 and 10 nm groups than in the CK group (*P*_5 nM_ < 0.001, *P*_10 nM_ < 0.001). Each group consisted of a total of 210 honeybees (3 independent cages × 70 honeybees per cage). (C) Schematic diagram of the five‐choice assay. Honeybees were housed in wooden cages equipped with five feeders containing sucrose solutions at varying concentrations of thiamethoxam (CK, SC, 1, 5, and 10 nm), and were free to move and select among the five feeders. (D) During the 10 days in the five‐choice assay, the daily feeding percentages of the thiamethoxam‐containing solutions were significantly higher than those of the CK solution (*p* < 0.001). Each group consisted of a total of 600 honeybees (3 independent cages × 200 honeybees per cage). Statistical analyses were conducted using two‐way ANOVA followed by Dunnett's post‐hoc tests for multiple comparisons [(B), (D)]. Significance levels are denoted as ^*^ *p* < 0.05, ^**^ *p* < 0.01, and ^***^ *p* < 0.001. Data were presented as mean ± SEM.

We next tested honeybee feeding preference for thiamethoxam under free‐choice conditions that better simulate natural foraging, where honeybees can autonomously select between different food sources. To simulate free‐living foraging conditions, honeybees were unrestrictedly housed in a wooden cage and simultaneously provided all five sucrose solutions (CK, SC, and thiamethoxam at concentrations of 1, 5, and 10 nm) in a five‐choice device (Figure 1C, see Section 4.5). Similar to the restricted laboratory conditions, honeybees showed a preference for thiamethoxam over 10 days, with this preference evident even from the first day. Daily feeding percentage and food intake for sucrose solutions containing thiamethoxam were significantly higher than in the CK group, showing a dose‐dependent increase (Figure 1D, *p* < 0.001; Figure S2A, *p* < 0.001). The preference index, which reflected honeybee food choice, further confirmed that sucrose solutions containing thiamethoxam had higher preference values, indicating that honeybees strongly preferred thiamethoxam‐laden food, even when provided with unrestricted access to alternative nontoxic food options (Figure S2B).

These results confirm that honeybees maintain a preference for thiamethoxam‐laden sucrose solutions even at lower than sublethal concentrations and a clean food source, that such preference is both rapid (within 1 day) and sustained (over 10 days), and that this preference is equally present in both more restricted and more natural laboratory conditions.

### 2.2 Honeybees May Not Detect Thiamethoxam‐Laden Food via Sensory Pathways

Next, we aimed to investigate how honeybees detect sucrose solutions containing thiamethoxam. Honeybee antennae are densely covered with various olfactory receptors [23], which may potentially mediate the detection of neonicotinoids.

To test this hypothesis, we used electroantennography (EAG) [24] to examine whether honeybee antennae could distinguish between various concentrations of thiamethoxam solutions based on volatile compounds [25] (see Section 4.6). EAG analysis was conducted on honeybees that had been exposed to different thiamethoxam‐laden solutions (CK, SC, and thiamethoxam at concentrations of 1, 5, and 10 nm) for 10 days prior to testing. Results showed no significant differences in EAG responses to thiamethoxam among all the tested groups (Figure 2A, *p* > 0.05), suggesting that thiamethoxam solutions lack volatile compounds [25] detectable by the olfactory receptors on honeybee antennae.

**FIGURE 2:**
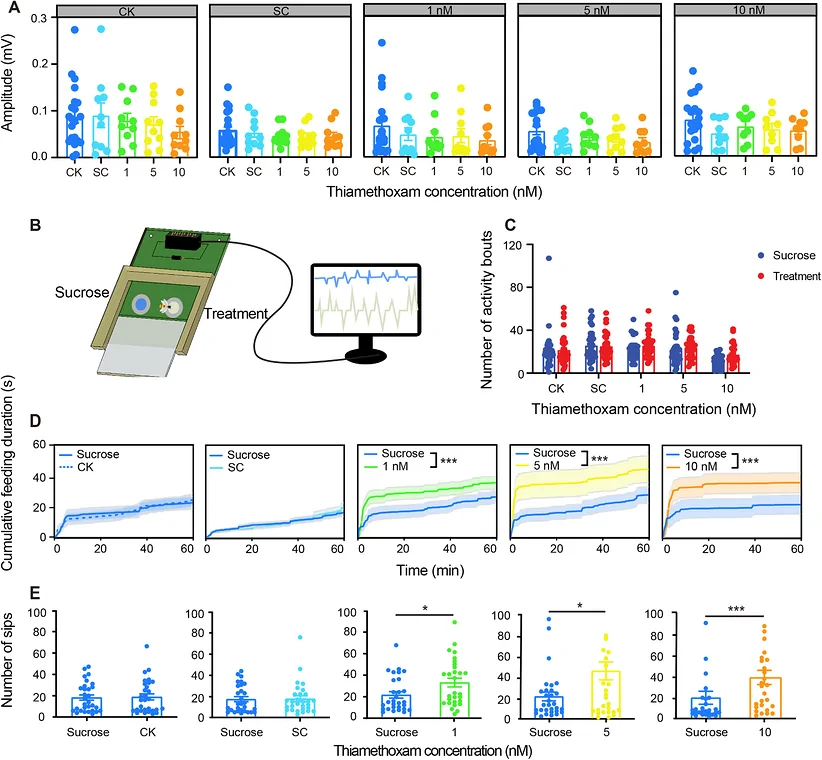
Honeybees cannot detect thiamethoxam and instead randomly select food sources. (A) Antennal electroantennography (EAG) amplitude did not show significant differences among groups (*p* > 0.05). The gray boxes indicate the feeding solutions, and labels below are the odor stimulus solutions used for EAG recording. Sample sizes: CK = 61, SC = 61, 1 nm = 61, 5 nm = 60, and 10 nm = 59. (B) Schematic diagram of the two‐choice assay. Honeybees were placed in the flyPAD chamber, where they could select between a sucrose solution and a sucrose solution containing a specific concentration of thiamethoxam. (C) There were no significant differences in the number of activity bouts among all groups (*p* > 0.05). *n* = 36 honeybees per group. (D,E) The cumulative feeding duration (D, *P*_1 nM_ < 0.001, *P*_5 nM_ < 0.001, *P*_10 nM_ < 0.001) and the number of sips (E, *P*_1 nM_ < 0.05, *P*_5 nM_ < 0.05, *P*_10 nM_ < 0.001) in the two‐choice assay of the thiamethoxam treatment groups were significantly higher than in the CK group. Sample sizes for D: *n* = 30 honeybees per group. Sample sizes for E: CK = 30, SC = 29, 1 nm = 29, 5 nm = 30, and 10 nm = 28. Statistical analyses were conducted using one‐way ANOVA followed by Dunnett's post‐hoc tests for multiple comparisons [(A)], two‐way ANOVA followed by Šídák's multiple comparisons test [(C)], and Wilcoxon matched‐pairs signed‐rank test [(D), (E)]. Significance levels are denoted as ^*^ *p* < 0.05, ^**^ *p* < 0.01, and ^***^ *p* < 0.001. Data were presented as mean ± SEM.

In addition to olfaction, honeybees also possess gustatory receptor neurons (GRNs) on their antennae and mouthparts. Previous studies have demonstrated that GRNs on the proboscis are more sensitive to toxins than those on the antennae or tarsi [26, 27, 28], and that GRNs in the proboscis do not respond to neonicotinoids, indicating that these chemicals in nectar or pollen cannot be perceived via gustatory pathways [10]. Together, these findings indicate that honeybees are unlikely to detect thiamethoxam‐laden food via either olfactory or gustatory pathways before ingestion. However, this raises a question: why do honeybees show a preference for thiamethoxam‐laden food? One possible explanation is that honeybees may initially select food sources during foraging due to the incapability of identifying thiamethoxam‐laden food; however, when honeybees accidentally consume thiamethoxam‐laden food, the thiamethoxam may induce positive feedback to honeybee physiology, thereby prompting honeybees to consume more thiamethoxam‐laden food, and thus forming the feeding preference.

To test whether this preference develops after consumption despite the lack of sensory detection, we used the flyPAD [29], an automated device for monitoring real‐time feeding behavior (Figure 2B). This system conducts a precise two‐choice assay with a high temporal resolution, making it possible to assess whether honeybees randomly or intentionally target certain food sources by analyzing the feeding duration and number of honeybees to a specific food. Honeybees were deprived of food for 2 h before the test and then individually placed in the flyPAD chamber, where each was allowed to choose between a control sucrose solution (CK or SC) and the three concentrations of thiamethoxam‐laden sucrose solutions (1, 5, and 10 nm; see Section 4.7). We recorded in detail the number of activity bouts, defined as the number of times the honeybee visited a specific food source, to determine whether their feeding behavior was random or targeted [29]. In addition, the cumulative time spent feeding and the number of sips were recorded to quantify feeding duration and intake frequency for a specific food [29]. Results showed no significant differences in the number of activity bouts between the sucrose group and any of the five treatments in the two‐choice assay (Figure 2C, *p* > 0.05), reconfirming that honeybees cannot detect thiamethoxam‐laden food. Nevertheless, both cumulative feeding duration and the number of sips for thiamethoxam solutions at 1, 5, and 10 nm were significantly higher than those of the sucrose group (Figure 2D, *P*_1 nM_ < 0.001, *P*_5 nM_ < 0.001, *P*_10 nM_ < 0.001; Figure 2E, *P*_1 nM_ < 0.05, *P*_5 nM_ < 0.05, *P*_10 nM_ < 0.001). These results support our hypothesis that honeybees randomly select food sources, yet still develop a preference for thiamethoxam‐laden food, which likely arises from positive feedback after consuming such food. However, how the positive feedback occurs requires further experimental validation.

### 2.3 Brain Dopamine Mediates Honeybee Preference for Thiamethoxam

Our results above support the viewpoint that honeybees develop a significant preference for thiamethoxam‐laden food, without the help of olfactory recognition, which is seemingly toxic but persistently consumed due to reward‐related reinforcement [30, 31], rather than an aversive or avoidance response. The reward system in honeybees shares several similarities with that of *Drosophila* [32, 33, 34], particularly involving neurotransmitters like dopamine [19, 35]. There is evidence showing that brain dopamine levels are elevated by most substances considered to be “addictive drugs”, and this elevation is also associated with food wanting in honeybees [19, 36, 37]. Thus, we hypothesized that thiamethoxam may trigger reward‐related dopamine signaling.

To investigate this hypothesis, we first conducted experiments to measure changes in brain dopamine levels in honeybees under resting conditions after 10 days of feeding exposure. Brain dopamine levels were measured using ultra‐high‐performance liquid chromatography‐tandem mass spectrometry (UHPLC‐MS/MS) in whole‐brain homogenates from honeybees after 10 days of feeding on either control (CK, SC) or thiamethoxam‐laden solutions (1, 5, and 10 nm; see Section 4.8). Results showed no significant differences in dopamine levels in the brains of honeybees across treatment groups (Figure 3A, *p* > 0.05), indicating that thiamethoxam exposure did not induce a sustained elevation of brain dopamine levels. However, does this mean that dopamine does not modulate the feeding preference of honeybees induced by thiamethoxam? A prior study demonstrated that transient activation of dopamine signaling mediates food wanting in honeybees [19]. Based on this finding, we further examined whether brain dopamine levels may transiently increase, rather than consistently be at a high level, to enhance the appetite of honeybees for thiamethoxam‐laden food.

**FIGURE 3:**
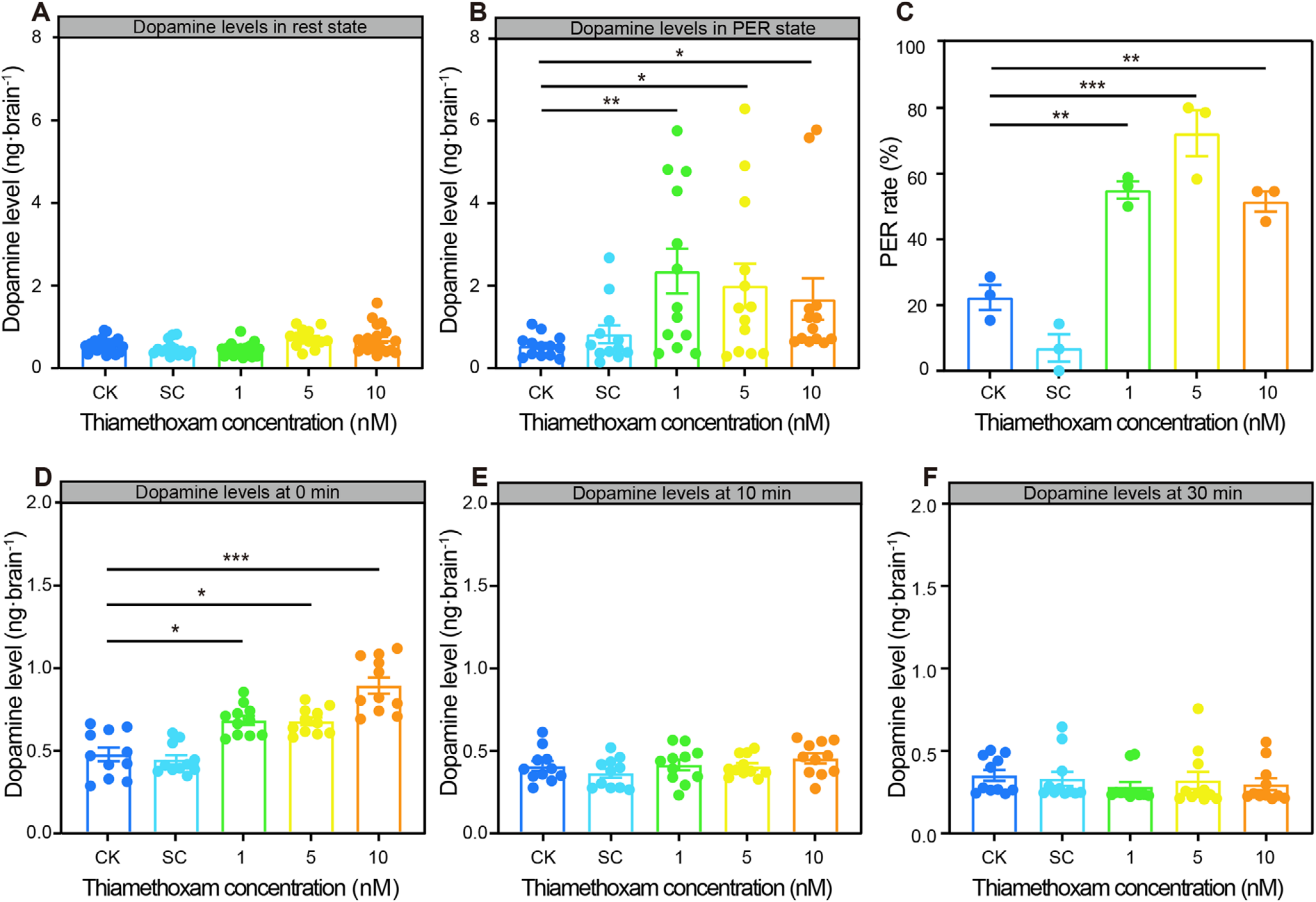
Brain dopamine is associated with feeding preference of honeybees for thiamethoxam. (A) In the resting state, there were no significant differences in the brain dopamine levels among all groups (*p* > 0.05). Sample sizes: CK = 20, SC = 15, 1 nm = 18, 5 nm = 14, and 10 nm = 20. (B) Among honeybees exhibiting a proboscis extension reflex (PER) to a sucrose‐free 1 nm thiamethoxam stimulus, brain dopamine levels in the thiamethoxam groups were significantly higher than in the CK group (*P*_1 nM_ < 0.01, *P*_5 nM_ < 0.05, *P*_10 nM_ < 0.05). Sample sizes: CK = 13, SC = 12, 1 nm = 13, 5 nm = 13, and 10 nm = 13. (C) The PER rates of honeybees in the thiamethoxam groups were significantly higher than in the CK group (*P*_1 nM_ < 0.01, *P*_5 nM_ < 0.001, *P*_10 nM_ < 0.01). Each group consisted of a total of 45 honeybees (3 independent cages × 15 honeybees per cage). The PER rate was calculated for each cage as the percentage of honeybees exhibiting PER among the 15 tested honeybees. (D–F) Brain dopamine dynamics during active feeding and after feeding. Dopamine levels were measured during active feeding (0 min; D), and at 10 min (E), and 30 min (F) after feeding. In the thiamethoxam treatment groups, dopamine levels were significantly elevated during active feeding compared to the CK group (D, *P*_1 nM_ < 0.05, *P*_5 nM_ < 0.05, *P*_10 nM_ < 0.001). Dopamine levels were no longer significantly different from the CK group at 10 min after feeding (E, *p* > 0.05), and remained not significantly different at 30 min after feeding (F, *p* > 0.05). Sample sizes: *n* = 11 per group per time point. For panels (A), (B), and (D–F), each biological replicate consisted of one individual honeybee brain. Statistical analyses were conducted using the Kruskal‐Wallis test followed by Dunn's post‐hoc tests for multiple comparisons [(A), (B), (D), (E), (F)], and one‐way ANOVA followed by Dunnett's post‐hoc tests for multiple comparisons [(C)]. Significance levels are denoted as ^*^ *p* < 0.05, ^**^ *p* < 0.01, and ^***^ *p* < 0.001. Data were presented as mean ± SEM.

To validate this hypothesis, after 10 days of feeding on CK, SC, and thiamethoxam at concentrations of 1, 5, and 10 nm, we measured brain dopamine levels in honeybees from all groups that exhibited the proboscis extension reflex (PER) to a sucrose‐free 1 nm thiamethoxam stimulus. Honeybee brains were immediately frozen in liquid nitrogen for subsequent analysis of dopamine levels (see Section 4.9 and 4.11). Results showed that brain dopamine levels and PER rates in the three thiamethoxam groups (1, 5, and 10 nm) were significantly higher than in the CK group (Figure 3B, *P*_1 nM_ < 0.01, *P*_5 nM_ < 0.05, *P*_10 nM_ < 0.05; Figure 3C, *P*_1 nM_ < 0.01, *P*_5 nM_ < 0.001, *P*_10 nM_ < 0.01), indicating an enhanced preference for thiamethoxam. To determine whether these dopamine changes reflected a broader alteration in brain biogenic amine levels, we further measured serotonin levels under both resting and PER states. Serotonin levels showed no significant differences among treatment groups in either the resting state or in honeybees exhibiting PER (Figure S3A,B, *p* > 0.05). These results suggest that brain dopamine, but not serotonin, is associated with thiamethoxam‐related PER responsiveness which may contribute to the observed preference of honeybees for thiamethoxam‐laden food.

However, the brain dopamine measurements under the PER state could not distinguish whether the observed dopamine elevation represented a transient or a sustained elevation. Therefore, we further examined brain dopamine dynamics during honeybee active feeding and after feeding. Honeybees from each treatment group were fed their corresponding treatment solutions, and brain dopamine levels were measured during active feeding (0 min) and at 10 and 30 min after feeding (see Section 4.10). The results showed that, during active feeding, brain dopamine levels in the thiamethoxam treatment groups were significantly higher than those in the CK group (Figure 3D, *P*_1 nM_ < 0.05, *P*_5 nM_ < 0.05, *P*_10 nM_ < 0.001). However, dopamine levels were no longer significantly different from the CK group at 10 min after feeding (Figure 3E, *p* > 0.05), or at 30 min after feeding (Figure 3F, *p* > 0.05). These results indicate that brain dopamine levels rapidly increased during active feeding, but this elevation was not sustained after feeding. Together with the resting and PER states findings, these results suggest that honeybee preference for thiamethoxam‐laden food is likely associated with transient activation of brain dopamine signaling [19], which is consistent with the dopamine modulatory mechanism of addiction‐like behaviors [31, 38, 39].

### 2.4 Blocking Honeybee Preference for Thiamethoxam

Given that brain dopamine levels are transiently increased in honeybees that exhibit a preference for thiamethoxam, we next tested whether blocking dopamine signaling could reduce this preference [40]. First, we conducted a five‐choice assay as described above (Figure 1C), but supplemented the three thiamethoxam solutions (1, 5, and 10 nm) with the dopamine receptor antagonist, fluphenazine (2 µg µL^−1^; see Section 4.12). This oral administration was used as targeted pharmacological interference of the thiamethoxam‐laden food options. Over the 10‐day exposure period, the feeding percentage and cumulative food intake for thiamethoxam‐laden food were both significantly lower than in the CK group (Figure 4A, *p* < 0.001; Figure 4B, *P*_1 nM_ < 0.001, *P*_5 nM_ < 0.001, *P*_10 nM_ < 0.001). However, given the bitter taste of fluphenazine, we questioned whether this observed feeding decrease may be attributed to gustatory aversion, rather than the blockade of dopamine.

**FIGURE 4:**
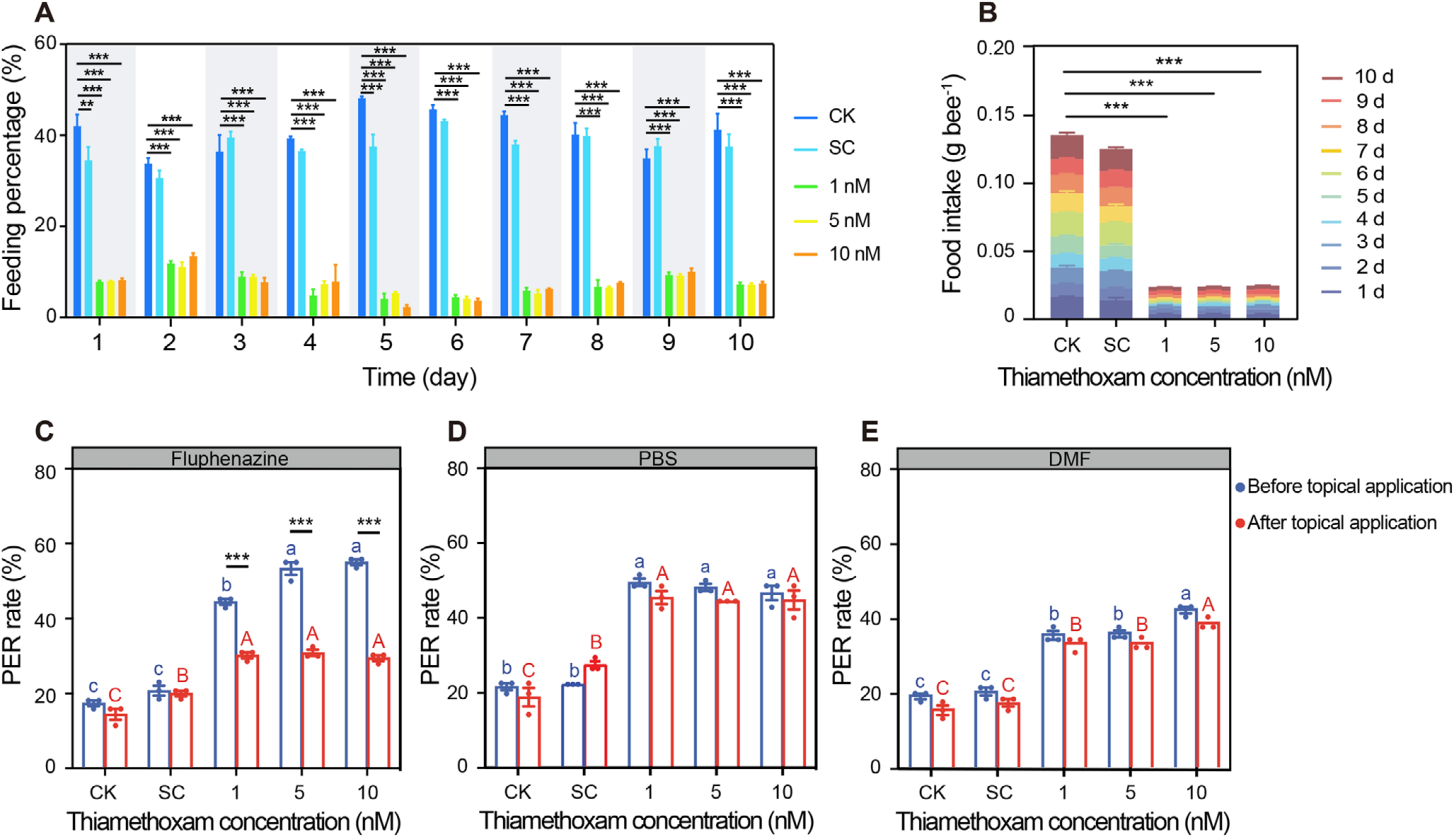
Blocking honeybee preference for thiamethoxam by interfering with dopamine signaling. (A,B) During the 10 days, the daily feeding percentage (A, *p* < 0.001) and cumulative food intake (B, *P*_1 nM_ < 0.001, *P*_5 nM_ < 0.001, *P*_10 nM_ < 0.001) of each solution in the thiamethoxam groups supplemented with fluphenazine were significantly lower than in the CK group. Each group consisted of a total of 450 honeybees (3 independent cages × 150 honeybees per cage). (C) Dopamine receptor antagonist fluphenazine significantly decreased PER rates of honeybees in thiamethoxam groups (*P*_1 nM_ < 0.001, *P*_5 nM_ < 0.001, *P*_10 nM_ < 0.001). Sample sizes for fluphenazine: CK = 44, SC = 45, 1 nm = 42, 5 nm = 40, and 10 nm = 48. (D,E) Neither phosphate buffer saline (PBS) (D) nor the solvent for fluphenazine N,N‐dimethylformamide (DMF) (E) treatments had a significant effect on the PER response to a sucrose‐free 1 nm thiamethoxam solution (*p* > 0.05). Sample sizes for PBS: CK = 31, SC = 44, 1 nm = 34, 5 nm = 45, and 10 nm = 42. Sample sizes for DMF: CK = 44, SC = 45, 1 nm = 42, 5 nm = 40, and 10 nm = 48. In panels (C–E), samples from each treatment group were randomly divided into three groups, and data points shown in the figure represent the mean of each group. The terms “Before topical application” refers to PER rates recorded before topical application of fluphenazine, PBS, or DMF, whereas “After topical application” refers to the PER rates measured again 30 min after application of the corresponding solution. Asterisks denote paired comparisons between “before” and “after” topical application within the same treatment group. Blue lowercase letters (a, b, c) indicate comparisons among treatment groups (CK, SC, 1, 5, and 10 nm) under the “before topical application” condition, whereas red uppercase letters (A,B,C) indicate comparisons among treatment groups under the “after topical application” condition. Statistical analyses were conducted using two‐way ANOVA with Dunnett's post‐hoc tests for multiple comparisons [(A), (B)], and one‐way ANOVA with Tukey's post‐hoc test was used to compare PER responses among the five groups in the “before” and “after” conditions separately, and two‐way ANOVA with Šídák's post‐hoc test was used to analyze within‐group differences between “before” and “after” time points [(C), (D), (E)]. Significance levels are denoted as ^*^ *p* < 0.05, ^**^ *p* < 0.01, and ^***^ *p* < 0.001. Data were presented as mean ± SEM.

To bypass the bitter taste of fluphenazine, rather than adding fluphenazine into the thiamethoxam‐laden food, we applied the dopamine receptor antagonist directly onto the thoraces of honeybees [19]. After the 10‐day oral exposure to control and thiamethoxam‐laden sucrose solutions, honeybees from each group were first tested for their PER response to 1 nm thiamethoxam (sucrose‐free) without introducing fluphenazine to establish a baseline feeding preference. Subsequently, fluphenazine was topically applied onto the thoraces of honeybees, and after a 30‐min waiting period for the antagonist to take effect, the PER response to 1 nm thiamethoxam was measured again (Section 4.13). Before topical application of fluphenazine, honeybees showed a preference for thiamethoxam‐laden food (Figure 4C), consistent with previous results (Figure 3C, *P*_1 nM_ < 0.01, *P*_5 nM_ < 0.001, *P*_10 nM_ < 0.01). After topical application of fluphenazine, PER rates of honeybees in the thiamethoxam groups significantly decreased (Figure 4C, *P*_1 nM_ < 0.001, *P*_5 nM_ < 0.001, *P*_10 nM_ < 0.001), indicating that dopamine blockade can effectively reduce the feeding preference of honeybees for thiamethoxam.

To exclude the possibility that merely applying a solution to the honeybee thorax somehow led to a change in PER, a control condition was added where a solution without fluphenazine was applied to the thorax. To test this, we used phosphate buffer saline (PBS) and N,N‐dimethylformamide (DMF) solutions with no fluphenazine added. The PER responses to a sucrose‐free 1 nm thiamethoxam were measured both before and 30 min after the topical application of either solution, following the same procedure as for fluphenazine to ensure a direct comparison of effects (see Section 4.13). Results showed that neither PBS nor DMF treatments had a significant effect on the PER rates to thiamethoxam solutions (Figure 4D, *p* > 0.05; Figure 4E, *p* > 0.05). Therefore, the observed decrease in PER rates (Figure 4C, *P*_1 nM_ < 0.001, *P*_5 nM_ < 0.001, *P*_10 nM_ < 0.001) was a result of the dopamine signaling blockade by fluphenazine.

Dopamine in honeybees is a multifunctional neuromodulator that extends well beyond hedonic food reward [19]. It has been implicated in aversive reinforcement learning [37, 41, 42], regulation of locomotor activity and arousal [43, 44], circadian rhythms [45], and division of labor and foraging specialization [46]. Consequently, broad application of dopamine antagonists may affect multiple dopaminergic pathways and cannot be interpreted as specifically associated with reward‐related circuits. Moreover, our behavioral and neurochemical measurements were not performed with circuit‐level resolution; dopamine was quantified from whole‐brain homogenates, which preclude distinction between dopamine synthesis, storage, and synaptic release. Thus, our findings support a role for dopamine in modulating insecticide‐related preference; they do not exclude broader effects arising from dopamine perturbation. Given the potential for increased insecticide exposure through dopamine‐modulated preference, future studies employing real‐time dopamine detection combined with feeding behavior monitoring will be crucial for establishing a direct link between dopamine dynamics and thiamethoxam‐related feeding behavior. Furthermore, our current study was conducted under laboratory conditions using cage‐reared worker honeybees, and therefore lacks the full social context of natural colonies (e.g., waggle dance recruitment and trophallaxis). We further state that future colony‐level studies should verify whether the dopamine‐associated thiamethoxam preference observed under laboratory conditions can be extended to colony‐level attraction or recruitment to contaminated nectar sources. Additionally, because the present study focused only on thiamethoxam, future studies should expand the testing to other neonicotinoids, such as imidacloprid, and to the major active metabolite of thiamethoxam, clothianidin, under environmentally relevant residue concentrations to determine whether similar dopamine‐associated preference responses can be induced by these compounds.

## 3 Conclusion

Our results demonstrate that honeybee preference for neonicotinoids may originate from enhanced appetitive motivation driven by transient activation of the dopamine system, and that blocking dopamine signaling by fluphenazine can effectively reduce this feeding preference. This association is correlative, and our measurements do not establish a causal link between dopamine activity and reward‐specific circuits. The risk posed by the neonicotinoid insecticide thiamethoxam lies in the fact that the preference of honeybees for insecticide‐laced solutions increases the likelihood of collecting contaminated nectar, thereby posing long‐term threats to colony health [10, 37]. Strategies based on modulation of the preference‐related dopamine pathway or developing insecticide formulations that are less attractive to honeybees could help reduce their exposure to insecticides and thus ensure stable pollination services in agricultural ecosystems.

## 4 Materials and Methods

### 4.1 Honeybees and Thiamethoxam Solutions

Honeybees (*Apis mellifera*) were obtained from the apiary located at the Institute of Apicultural Research, Chinese Academy of Agricultural Sciences, Beijing (N39°59′35.33″, E116°11′59.74″) [47]. The experiment was conducted from May 2023 to August 2026. Prior to the formal experiment, five colonies were pre‐screened to avoid the interference of major parasites and pathogens. Three colonies that met the negative screening criteria for no major parasites (*Varroa* *destructor* and *Nosema* spp.), common viruses (deformed wing virus, DWV; black queen cell virus, BQCV), and bacterial pathogens (American foulbrood, AFB; European foulbrood, EFB) were selected as the source colonies (see Section 4.2). Three capped‐brood frames from three different colonies were transported to the laboratory and incubated in an incubator (RXZ–380, Ningbo, China) at 35°C and 60% relative humidity for 24 h, after which newly emerged worker honeybees were collected, placed in wooden cages (12 cm × 6 cm × 10 cm) with mesh on two sides, and reared in the incubator (30°C and 50% relative humidity) for 10 days.

A stock solution of thiamethoxam (7924–100MG–R, Sigma‐Aldrich, USA) was prepared by dissolving 10 mg powder in 0.343 mL of dimethyl sulfoxide (DMSO) to create a 1 × 10^8^ nm concentration. The solution was then aliquoted and stored in a −20°C freezer in the dark until use. Before administration, it was diluted in 50% (w/v) sucrose solution to achieve final thiamethoxam concentrations of 1 nm (0.29 ng g^−1^), 5 nm (1.46 ng g^−1^), 10 nm (2.92 ng g^−1^), 15 nm (4.38 ng g^−1^), 30 nm (8.76 ng g^−1^), and 40 nm (11.68 ng g^−1^) for each treatment group. An additional control group (CK) received only 50% (w/v) sucrose solution, while the solvent control (SC) contained the same DMSO concentration as the 40 nm thiamethoxam treatment group, resulting in a final DMSO concentration of 0.00004% (v/v) in the sucrose solution. For the proboscis extension reflex (PER) assay described below, a sucrose‐free 1 nm thiamethoxam stimulus solution was prepared separately by diluting the stock solution in distilled water. All solutions were freshly prepared each day and protected from light throughout experiments.

### 4.2 Pathogen Screening of Honeybee Colonies

Five candidate honeybee colonies were initially screened for major parasites and pathogens, and three colonies that met the negative screening criteria were selected for the experiments. *Varroa destructor* infestation was assessed by visual inspection and the powdered sugar shake method. Colonies with an average mite count of less than 0.1 mites per 100 honeybees were considered to have no detectable *Varroa destructor* infestation. *Nosema* spp. infection was examined by microscopic observation of homogenized midgut tissues from ten worker honeybees per colony, and samples without detectable spores were considered negative. For viral screening, a pooled sample of five worker honeybees from each colony was used for total RNA extraction, reverse transcription, and PCR amplification with virus‐specific primers to detect DWV and BQCV. For bacterial pathogen screening, pooled samples of five worker honeybees from each colony were used for total DNA extraction and PCR amplification with pathogen‐specific primers to detect AFB and EFB. The absence of target bands was considered negative. Detailed primer sequences, reaction conditions, and gel electrophoresis results are provided in Tables S1–S3 and Figure S4.

### 4.3 Physiological Experiment

To assess the toxic effects of different thiamethoxam concentrations (1, 5, 10, 15, 30, and 40 nm) on honeybee physiological parameters, survival and body weight were monitored daily over a 10‐day exposure period. Each group had three cages, and each cage contained 70 honeybees. Survival was recorded as the proportion of alive honeybees in the total honeybees each day. The body weight of honeybees in each group was also recorded daily. Six honeybees were randomly selected from each cage every day, and their mean body weight was measured.

### 4.4 Fixed‐Choice Feeding Preference Assay

Newly emerged worker honeybees were housed in cages, and each cage was provided exclusively with one of five solutions: CK, SC, or thiamethoxam (1, 5, and 10 nm). Each group had three cages, and each cage contained 70 honeybees. No pollen was provided during the experimental period. All solutions were confirmed in physiological tests to have no significant negative impact on honeybee health. The food intake per honeybee was calculated by measuring the total sucrose solution consumed in each cage and dividing it by the number of surviving honeybees, which was monitored over 10 days. An additional cage without honeybees was used to measure evaporation of the feeding solution.

### 4.5 Five‐Choice Feeding Preference Assay

Newly emerged worker honeybees were housed in a wooden cage (38 cm × 34 cm × 16 cm), and they were simultaneously provided with five sucrose solutions: CK, SC, and thiamethoxam at concentrations of 1, 5, and 10 nm. As in the fixed‐choice assay, no pollen was provided during the experimental period. The assay included three cages, and each cage contained 200 honeybees. Honeybees were allowed to freely select among the five food options for 10 days, with the position of the solutions kept constant throughout the experiment. We conducted daily monitoring of the food intake from each feeder over 10 days. The food intake was calculated using the method described in Section 4.4, and the feeding percentage was determined by dividing the amount of sucrose solution consumed from an individual feeder by the total consumption of the five feeders. Furthermore, we performed a preference index analysis according to the formula: (treatment group—control group) / (treatment group + control group). Food intake was recorded at the cage level; therefore, individual‐level intake and possible trophallactic food exchange among cage‐mates could not be separately quantified.

### 4.6 Electroantennography

Honeybees were randomly selected from each group for electroantennography (EAG) recording after being fed CK, SC, or thiamethoxam (1, 5, or 10 nm) for 10 days. Honeybees were immobilized on ice, and either the left or right antennae were excised at the base using a scalpel. The excised antennae were then clamped between two distal electrodes with conductive gel (REF15–60, Sigma, USA). Odor stimuli included CK, SC, thiamethoxam at concentrations of 1, 5, and 10 nm, and a positive control (1% hexanal diluted in mineral oil). Each stimulus was applied using 10 µL of solution on a 0.5 cm × 2 cm filter paper strip. Odors were delivered via a continuous airflow at 20 mL s^−1^. Each stimulus was presented for 0.5 s, followed by a 30‐second interval between stimulations to ensure baseline restoration, and repeated three times. The antennae amplitudes were recorded as changes in electrical potential [48]. EAG measurements were performed with an Intelligent Data Acquisition Controller (IDAC–2–USB, Syntech, Germany) at a sample rate of 200 Hz, with signals filtered using a 10 Hz high‐pass filter. Data were analyzed offline using Gc‐Ead 1.2.5.

### 4.7 Two‐Choice Thiamethoxam Discrimination Assay

The feeding behavior of honeybees was monitored using the flyPAD device after a 10‐day exposure to CK, SC, or thiamethoxam at concentrations of 1, 5, and 10 nm. The flyPAD is an automated, high‐throughput system that detects feeding amounts by measuring capacitance changes [29]. There were 36 honeybees in each group. Each flyPAD chamber had two channels with circular metal pads with a hollow center holding the liquid food: the control sucrose solution was placed on the left, while the right channel contained either one of the two controls (CK, SC) or one of the thiamethoxam at concentrations of 1, 5, and 10 nm. Honeybees were starved for 2 h before being placed in the flyPAD chamber, where they could move and feed freely on either solution for 1 h. Honeybee movement around the metal pad of each channel, as well as feeding activity, was converted into electrical signals and recorded. Data analysis included three parameters: (i) number of activity bouts, representing the number of times honeybees visited a specific food source; (ii) cumulative feeding duration, representing active feeding time; and (iii) number of sips, representing the frequency of feeding actions.

### 4.8 Dopamine and Serotonin Levels in Resting Honeybee Brains

To further examine the role of dopamine in the feeding preference behavior of honeybees, honeybees were randomly selected from CK, SC, and thiamethoxam at concentrations of 1, 5, and 10 nM groups after 10 days of feeding, and immediately frozen in liquid nitrogen. Resting honeybees were defined as honeybees sampled without any external stimulation, and food sources were removed before sampling to avoid active feeding. The hypopharyngeal glands, compound eyes, ocelli, and other glandular tissues were removed on dry ice using sterile tweezers. Subsequently, the whole brain was dissected and collected as one intact brain sample. The brain sample was immediately frozen in liquid nitrogen and stored at −80°C until analysis.

Dopamine and serotonin levels in individual whole honeybee brains were analyzed using ultra‐high‐performance liquid chromatography‐tandem mass spectrometry (UHPLC‐MS/MS) (Thermo Fisher Scientific, Waltham, USA). After the addition of 200 µL cold buffer (0.4 mol L^−1^ perchloric acid, 2.6 mm Na_2_O_5_S_2_, and 2.7 mm EDTA), samples were homogenized using a high‐throughput tissue grinder (CGRINDER–24) and subjected to ultrasonic treatment in cold water for 20 min. The homogenate was then transferred to a 1.5 mL microcentrifuge tube, filtered using a 0.22 µm filter membrane, and centrifuged at 9000 rpm at 4°C for 5 min. The filtrate was transferred to a microvial for UHPLC‐MS/MS analysis. Each sample was injected at a volume of 2 µL into the XBridge BEH C18 column (2.5 µm, 2.1 mm × 150 mm) (Agilent, Santa Clara, USA) maintained at 35°C. Eluent A consisted of a 0.1% formic acid solution in water, and eluent B was acetonitrile. The eluent gradient changed according to the following conditions: 0 ∼ 3 min, 95% A; 3 ∼ 4.5 min, 95% ∼ 60% A; 4.5 ∼ 5.5 min, 60% A; 5.5 ∼ 7.5 min, 60% ∼ 10% A; 7.5 ∼ 10 min, 10% ∼ 95% A; 10 ∼ 13 min, 95% A (flow rate: 0.3 mL min^−1^). The standard curve was redrawn before each analysis, and the dopamine and serotonin levels in each brain tissue sample were determined by comparing the peak area to the standard curve. Data collection and analysis of the UHPLC‐MS/MS results were carried out using MassHunter software (Agilent, Santa Clara, USA).

### 4.9 Brain Dopamine and Serotonin Levels in Honeybees Exhibiting PER

On day 10, honeybees were randomly selected from the CK, SC, and thiamethoxam treatment groups at concentrations of 1, 5, and 10 nm. To measure brain dopamine and serotonin levels in honeybees exhibiting a PER in response to thiamethoxam stimulation, honeybees were anesthetized on ice and then mounted in custom‐made plastic tubes; the antennae of honeybees were stimulated with 1 µL of sucrose‐free 1 nm thiamethoxam solution prepared in distilled water. Honeybees exhibiting PER were immediately frozen in liquid nitrogen. This sampling criterion was applied to all groups, including the CK group. These honeybees did not actually consume the stimulus solution. Subsequently, the dissection was performed as described in Section 4.8, and UHPLC‐MS/MS was used to determine the dopamine and serotonin levels in the honeybee brains.

### 4.10 Brain Dopamine Levels in Honeybees During and After Feeding

To examine dopamine dynamics during and after feeding, three sampling time points were set: during active feeding (0 min), 10 min after feeding, and 30 min after feeding. After the exposure period, honeybees were randomly selected from each treatment group (CK, SC, and thiamethoxam at concentrations of 1, 5, and 10 nm) and mounted in custom‐made plastic tubes. Each selected honeybee was fed 5 µL of its corresponding treatment solution. For the 0 min time point, honeybees were immediately frozen in liquid nitrogen while actively feeding. For the 10 and 30 min time points, honeybees were allowed to consume the 5 µL solutions and were then frozen in liquid nitrogen at 10 or 30 min after feeding, respectively. Each treatment group included 11 honeybees per time point, with each individual brain used as one biological replicate. Brain tissues were subsequently dissected, and dopamine levels were measured by UHPLC‐MS/MS.

### 4.11 PER of Honeybees for Thiamethoxam

Honeybees were exposed to different treatment solutions (CK, SC, or thiamethoxam at concentrations of 1, 5, and 10 nm) for 10 days. To evaluate whether pre‐exposure would increase honeybee feeding preference for thiamethoxam‐laden food, we performed the PER assay after the 10‐day exposure period. For the assay, honeybees were anesthetized on ice and then mounted in custom‐made plastic tubes, which restricted body movement while allowing free movement of the antennae and mouthparts. Honeybee antennae were stimulated with 1 µL of sucrose‐free 1 nm thiamethoxam solution using a micropipette for 2 s to record the PER. Each treatment group consisted of 45 honeybees from 3 independent cages, with 15 honeybees tested per cage.

### 4.12 Blocking Honeybee Preference by Oral Exposure to Fluphenazine

To determine whether a dopamine receptor antagonist affects the feeding preference of honeybees, we employed a similar setup to the previous five‐choice assay. The five solutions included CK, SC, and thiamethoxam at concentrations of 1, 5, and 10 nm. The difference was that thiamethoxam solutions (1, 5, and 10 nm) were supplemented with the dopamine receptor antagonist fluphenazine (HY–A0081, SparkJade, China) [49]. Fluphenazine, a dopamine receptor antagonist in honeybees [33, 34], was used at a concentration of 2 µg µL^−1^ dissolved in the fluphenazine solvent N,N‐dimethylformamide (DMF) (68–12–2, Sigma–Aldrich, USA). Honeybees were allowed to freely select among the solutions for 10 days, and the feeding percentage and food intake were measured using the method described in Section 4.5 to evaluate changes in honeybee preference. Each group had three cages, and each cage contained 150 honeybees.

### 4.13 Blocking Honeybee Preference by Topically Applying Fluphenazine

To eliminate the uncertainty in the results caused by the bitterness of fluphenazine, we further investigated whether blocking dopamine could reduce honeybee preference for thiamethoxam. After 10 days of exposure to different treatments (CK, SC, or thiamethoxam at concentrations of 1, 5, and 10 nm), 1 µL of fluphenazine solution was topically application onto the thorax of each honeybee, following a previously reported method [19, 48]. The PER responses of honeybees to the 1 nm thiamethoxam solution were recorded before fluphenazine application, and their PER responses were re‐evaluated and recorded 30 min after fluphenazine application.

To exclude the potential impact of the applied procedure and solvent effects, phosphate buffer saline (PBS) and DMF were applied onto the thoraces of honeybees treated with different thiamethoxam concentrations, and their PER response to a 1 nm thiamethoxam solution (sucrose‐free) was measured before and after the applications.

### 4.14 Statistics

Data were tested for normality using the Shapiro–Wilk test. The survival of honeybees was analyzed using the log‐rank (Mantel‐Cox) test (Figure S1A). The electroantennography responses to different solutions, PER rate and body weight were analyzed using one‐way ANOVA followed by Dunnett's post‐hoc tests for multiple comparisons (Figures 2A and 3C and Figure S1B). The analysis of honeybee preference behavior during feeding on neonicotinoid‐laden food was analyzed by two‐way ANOVA followed by Dunnett's post‐hoc tests for multiple comparisons (Figures 1B,D and 4A,B and Figure S2A). In the two‐choice test, behavior data were analyzed using two‐way ANOVA with Šídák's post‐hoc test to compare the number of activity bouts (Figure 2C). The cumulative feeding duration and the number of sips were compared by Wilcoxon matched‐pairs signed rank test (Figure 2D,E), and the Kruskal‐Wallis test with Dunn's post‐hoc tests for multiple comparisons (Figure 3A,B,D–F and Figure S3). To further verify if blocking dopamine could reduce honeybee preference, one‐way ANOVA with Tukey's post‐hoc test was used to compare PER responses among the five groups in the “before” and “after” conditions separately, and two‐way ANOVA with Šídák's post‐hoc test was used to analyze within‐group differences between “before” and “after” time points (Figure 4C–E). Statistical analyses and data visualization were performed using GraphPad Prism 9 (GraphPad Software, USA). Statistical significance was set at *p* < 0.05.

## Author Contributions

Conceptualization: YG, YL. Methodology: JL, YG, WF, YL. Investigation: JL, WF. Visualization: JL, YL. Funding Acquisition: YG, YL. Project Administration: JL, YL. Supervision: YG, YL. Writing – original Draft: JL, YG, YL. Writing – review & Editing: JL, YG, WF, DL, PD, YL.

## Funding

This work was supported by the Chinese Academy of Agricultural Sciences (Elite Youth Program to Y.L.) and the Chinese Academy of Sciences (SKLURE2022‐1–3).

## Conflicts of Interest

The authors declare no conflicts of interest.

## Supporting information




**Supporting File**: advs78535‐sup‐0001‐SuppMat.docx.

## Data Availability

The data that support the findings of this study are available from the corresponding author upon reasonable request.
