# Supplementary material for: Blocking Honeybee Preference for Neonicotinoids by Interfering With Dopamine Signaling
Source: Adv Sci (Weinh). 2026 Nov 4:e78535. Online ahead of print. doi: 10.1002/advs.78535 (PMC13736161; doi:10.1002/advs.78535)
Supplement: Supplementary file 1 [file ADVS-9999-e78535-s001.docx]

**Supplementary information**

**Blocking honeybee preference for neonicotinoids by interfering with dopamine signaling**

Jing Li^1,2,3^, Yuan Ge^2,3*^, Wangjiang Feng^1^, David C. Lyon^4^, Pingli Dai^1^, Yong-Jun Liu^1*^

^1^State Key Laboratory of Resource Insects, Institute of Apicultural Research, Chinese Academy of Agricultural Sciences; Beijing, China

^2^State Key Laboratory of Regional and Urban Ecology, Research Center for Eco-Environmental Sciences, Chinese Academy of Sciences; Beijing, China

^3^University of Chinese Academy of Sciences; Beijing, China

^4^Department of Anatomy and Neurobiology, School of Medicine, University of California; Irvine, California, USA

***Correspondence**: Yuan Ge ([yuange@rcees.ac.cn);](mailto:yuange@rcees.ac.cn;) Yong-Jun Liu (liuyongjun@caas.cn)

**The file includes:**

**Figure S1–4**

**Table S1–3**

**Figure S1. Higher concentrations of thiamethoxam decrease the survival and body weight of honeybees. (A)** Thiamethoxam (>10 nM) significantly decreased honeybee survival (*P*_15 nM_ < 0.01, *P*_30 nM_ < 0.001, *P*_40 nM_ < 0.001). No significant differences were observed between the CK and SC groups in survival (*P*_SC_ > 0.05). Each group consisted of a total of 210 honeybees (3 independent cages × 70 honeybees per cage). **(B)** Compared with the CK group, thiamethoxam at concentrations of 40 nM significantly decreased honeybee body weight (*P*_40 nM_ < 0.05). No significant differences were observed between the CK and SC groups in body weight (*P*_SC_ > 0.05). Six honeybees were randomly selected from each cage each day, and their mean body weight was used as one replicate. Statistical analyses were conducted using the log-rank (Mantel-Cox) test [(A)], and one-way ANOVA followed by Dunnett’s post-hoc tests for multiple comparisons [(B)]. Significance levels were denoted as * *p* < 0.05, ** *p* < 0.01, and *** *p* < 0.001. Data were presented as mean ± SEM.

**Figure S2. Honeybees prefer thiamethoxam-laden food. (A)** During the 10 days in the five-choice assay, the daily food intake of honeybees in the thiamethoxam groups was significantly higher than in the CK group (*p* < 0.001). **(B**) During the 10 days in the five-choice assay, honeybees showed a higher preference index for thiamethoxam solutions (1, 5, and 10 nM). There were three independent cages, with 200 honeybees per cage included in the assay. Statistical analyses were conducted using two-way ANOVA followed by Dunnett’s post-hoc tests for multiple comparisons [(A)]. Significance levels were denoted as * *p* < 0.05, ** *p* < 0.01, and *** *p* < 0.001. Data were presented as mean ± SEM.

**Figure S3. Brain serotonin levels were not affected by thiamethoxam exposure. (A)** In the resting state, there were no significant differences in brain serotonin levels among all groups (*p* > 0.05). **(B)** In the state exhibiting a proboscis extension reflex (PER), there were no significant differences in brain serotonin levels among all groups (*p* > 0.05). Each group included 11 biological replicates, with each replicate consisting of one individual honeybee brain. Statistical analyses were conducted using the Kruskal-Wallis test followed by Dunn's post-hoc tests for multiple comparisons [(A), (B)]. Data were presented as mean ± SEM.

**Figure S4. Pathogen screening of honeybee colonies. (A)** Microscopic examination of *Nosema* spp. spores in midgut tissues of worker honeybees at 400× magnification. The first panel shows a positive control, with a red arrow indicating a *Nosema* spp. spore, whereas Colonies 1–3 show no detectable spores. (**B**) PCR amplification results for deformed wing virus (DWV) and black queen cell virus (BQCV). The left panel shows the positive control, whereas Colonies 1–3 show no target bands. (**C**) PCR amplification results for American foulbrood (AFB) and European foulbrood (EFB). The left panel was the positive control, whereas Colonies 1–3 show no target bands. For *Nosema* spp. detection, 10 worker honeybees per colony were examined; for viral and bacterial pathogen detection, pooled samples of 5 worker honeybees per colony were used.
**Table S1. Primer sequences used for pathogen detection.**

| **Target pathogen** | **Primer sequences (5’–3’)** | **Amplicon (bp)** |
| --- | --- | --- |
| Deformed wing virus (DWV) | F: TACTAGTGCTGGTTTTCCTTT | 155 |
|  | R: CTCATTAACTGTGTCGTTGAT |  |
| Black queen cell virus (BQCV) | F: TGGTCAGCTCCCACTACCTTAAAC | 700 |
|  | R: GCAACAAGAAGAAACGTAAACCAC |  |
| American foulbrood (AFB) | F: GCTCTGTTGCCAAGGAAGAA | 451 |
|  | R: AGGCGGAATGCTTACTGTGT |  |
| European foulbrood (EFB) | F: GAAGAGGAGTTAAAAGGCGC | 831 |
|  | R: TTATCTCTAAGGCGTTCAAAGG |  |

**Table S2. Thermal cycling conditions for PCR amplification.**

| **Step** | **Temperature** | **Time** | **Cycles** |
| --- | --- | --- | --- |
| Initial denaturation | 94℃ | 2 min | 1 |
| Denaturation | 94℃ | 30 s | 35 |
| Annealing | 55℃ |  |  |
| Extension | 72℃ |  |  |
| Final extension | 72℃ | 5 min | 1 |
| Hold | 4℃ | ∞ | — |

**Table S3. Reverse transcription reaction system.**

| **Component** | **Volume (μL)** | **Final concentration** |
| --- | --- | --- |
| dNTP Mix (2.5 mM each) | 4 | 500 μM each |
| Primer Mix | 2 | — |
| RNA Template | 1 | 50 pg–5 μg |
| 5 × RT Buffer | 4 | 1× |
| DTT (0.1 M) | 2 | 10 mM |
| HiFiScript Reverse Transcriptase (200 U/μL) | 1 | — |
| RNase-Free Water | Up to 20 | — |
| Total | 20 |  |
